# A Novel 3D‐Printed Strut‐Type Prosthesis Reconstructs the Hemicortical Defect After Distal Femur Parosteal Osteosarcoma Resection: The Mid‐Term Follow‐Up Outcome

**DOI:** 10.1111/os.70360

**Published:** 2026-06-18

**Authors:** Jianhua Mu, Yitian Wang, Han Liu, Xuanhong He, Zhuangzhuang Li, Minxun Lu, Fan Tang, Yi Luo, Yong Zhou, Li Min, Chongqi Tu

**Affiliations:** ^1^ Department of Orthopedics Orthopeadic Research Institute, West China Hospital, West China Medical School, Sichuan University Chengdu Sichuan China; ^2^ Model Worker and Craftsman Talent Innovation Workshop of Sichuan Province West China Hospital, Sichuan University Chengdu Sichuan China

**Keywords:** 3D‐printed prosthesis, hemicortical resection, metaphysis of the distal femur, parosteal osteosarcoma, reconstruction

## Abstract

**Background:**

Parosteal osteosarcoma (PAOS) is a rare and low‐grade primary bone malignant tumor that typically occurs in the distal femur. Traditional reconstruction options for distal femur PAOS include distal femoral replacement (DFR), autografts, and allografts, but issues such as functional sacrifice, insufficient mechanical strength, and low bone union rate limit their further clinical application. 3D‐printed prosthesis, due to the matching shape of various bone defects, could be a novel strategy to reconstruct the hemicortical defect after distal femur PAOS tumor resection. In this study, we designed a 3D‐printed strut‐type prosthesis (3DPSP) to repair the hemicortical defect and evaluated the mid‐term effect in distal femur PAOS.

**Methods:**

A retrospective analysis was conducted on patients diagnosed with PAOS at the distal femur between April 2018 and January 2021. Six patients received DFR and six patients received customized 3DPSP reconstruction. Clinical outcomes including oncology status, defect lengths, and ranges of motion of the knee, Musculoskeletal Tumor Society (MSTS) scores, and complications were recorded. X‐ray was performed to evaluate the position and stability of the prosthesis and T‐SMART was applied to assess the interface osteointegration between prosthesis and bone.

**Results:**

A total of 12 patients were enrolled in this study with an average follow‐up of 42.1 ± 11.8 months, ranging from 22 to 59 months. In the 3DPSP group, the average operation time was 2.3 ± 0.4 h; the intraoperative blood loss was 175.0 ± 82.2 mL. The average range of motion of the knee was 0°–123° and the mean MSTS score was 28.3 ± 1.6. Compared to the DFR group, the 3DPSP group exhibited significantly less intraoperative blood loss and superior functional recovery.

**Conclusions:**

In this study, we designed a novel 3DPSP to reconstruct the hemicortical defect after distal femur PAOS resection. The mid‐term follow‐up result demonstrated that this prosthesis could be an effective reconstruction option for distal femur PAOS patients because of the restored limb function and favorable bone‐prosthesis integration. Further observation of this novel prosthesis is needed.

## Graphical abstract

This study developed a novel three‐dimensional (3D)‐printed strut‐type prosthesis for reconstruction of bone defects following hemicortical resection of distal femoral parosteal osteosarcoma. The mid‐term follow‐up results demonstrated that this prosthesis effectively restored limb function and exhibited favorable bone ingrowth and osseointegration.

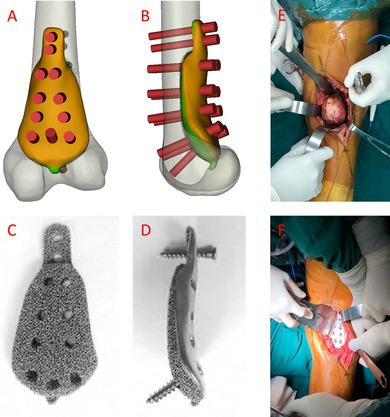

## 1 Introduction

Parosteal osteosarcoma (PAOS) is a rare primary bone tumor, comprising 3%–6% of osteosarcomas and predominantly occurring in young females aged 20–40 years [1, 2, 3, 4]. The posterior distal femoral metaphysis is mostly involved [5]. Current mainstream treatment methods primarily consist of prosthetic reconstruction following segmental tumor resection, as well as biological reconstruction after hemicortical resection [6]. Endoprosthetic distal femur replacement (DFR) following en bloc resection has exhibited favorable long‐term survival rates [7, 8]. However, the natural knee joint and affiliated soft tissue are inevitably sacrificed to obtain a safe resection margin. Meanwhile, a higher rate of revision due to postoperative complications, including infection, aseptic loosening, and mechanical failure was also reported [9]. Given the low‐grade malignancy of PAOS and advancements in chemotherapy and surgical techniques, partial cortical resection has been sufficient for some patients to achieve safe resection margins. In this case, biological reconstruction including autografts and allografts implants with plates and screws fixation was applied to preserve as much normal bone and soft tissue as possible, offering biocompatibility and mechanical support. However, complications such as bone resorption, nonunion, disease transmission, and infection potentially necessitating further surgical interventions have limited their further application [10, 11]. Additionally, the delayed postoperative immobilization due to the low mechanical strength may cause more complications such as embolism and poor functional recovery [12].

Recently, 3D printing technology has been introduced into orthopedics, bringing numerous advantages such as personalized customization and porous interfaces [13, 14, 15, 16, 17, 18, 19]. Furthermore, many studies reported that the application of custom‐made porous prostheses offers highly matched shapes for bone defect and promotes excellent osseointegration [20, 21]. Nonetheless, the application of 3D‐printed prostheses to hemicortical reconstruction in distal femoral PAOS remains limited. To address this gap, the present study was designed to develop a patient‐specific 3D‐printed strut‐type prosthesis (3DPSP) with a porous titanium surface. We aimed to evaluate (i) the mid‐term oncological outcomes of this joint‐preserving reconstruction strategy, including implant survival and local tumor control, (ii) postoperative functional outcomes, including bone–prosthesis integration, knee range of motion, MSTS scores, and complication rates, and (iii) its perioperative and early functional advantages compared with conventional DFR, particularly regarding operative time, intraoperative blood loss, and early rehabilitation.

## 2 Methods

### 2.1 Patient Demographics

This retrospective study was approved by the institutional review board. Between April 2018 and January 2021, 12 patients diagnosed with PAOS of the distal femoral metaphysis were screened according to the following inclusion criteria: (a) histopathologically confirmed low‐grade PAOS; (b) primary tumor located in the metaphysis of the distal femur without prior surgical treatment at the affected site; (c) surgical treatment consisting of either hemicortical resection and reconstruction with a customized 3DPSP or DFR with a modular endoprosthesis; (d) complete preoperative imaging records and baseline data; and (e) a minimum postoperative follow‐up of 12 months. Exclusion criteria were: (a) severe comorbidity or poor performance status; (b) synchronous multiple primary malignancies; and (c) radiological images with significant artifacts that prevented accurate assessment of tumor extent or prosthesis integration. Patients who received biological reconstruction were not included in this comparative cohort. The 3DPSP reconstruction was indicated for patients with low‐grade PAOS whose tumors were confined to the metaphyseal region, did not extend into the epiphysis or articular surface, and involved ≤ 50% of the cortical circumference, allowing hemicortical excision to achieve wide surgical margins while preserving the native knee joint and cruciate ligaments. DFR was indicated for tumors with extensive epiphyseal involvement or larger lesions that required complete articular surface resection to obtain safe oncological margins.

**FIGURE 1:**
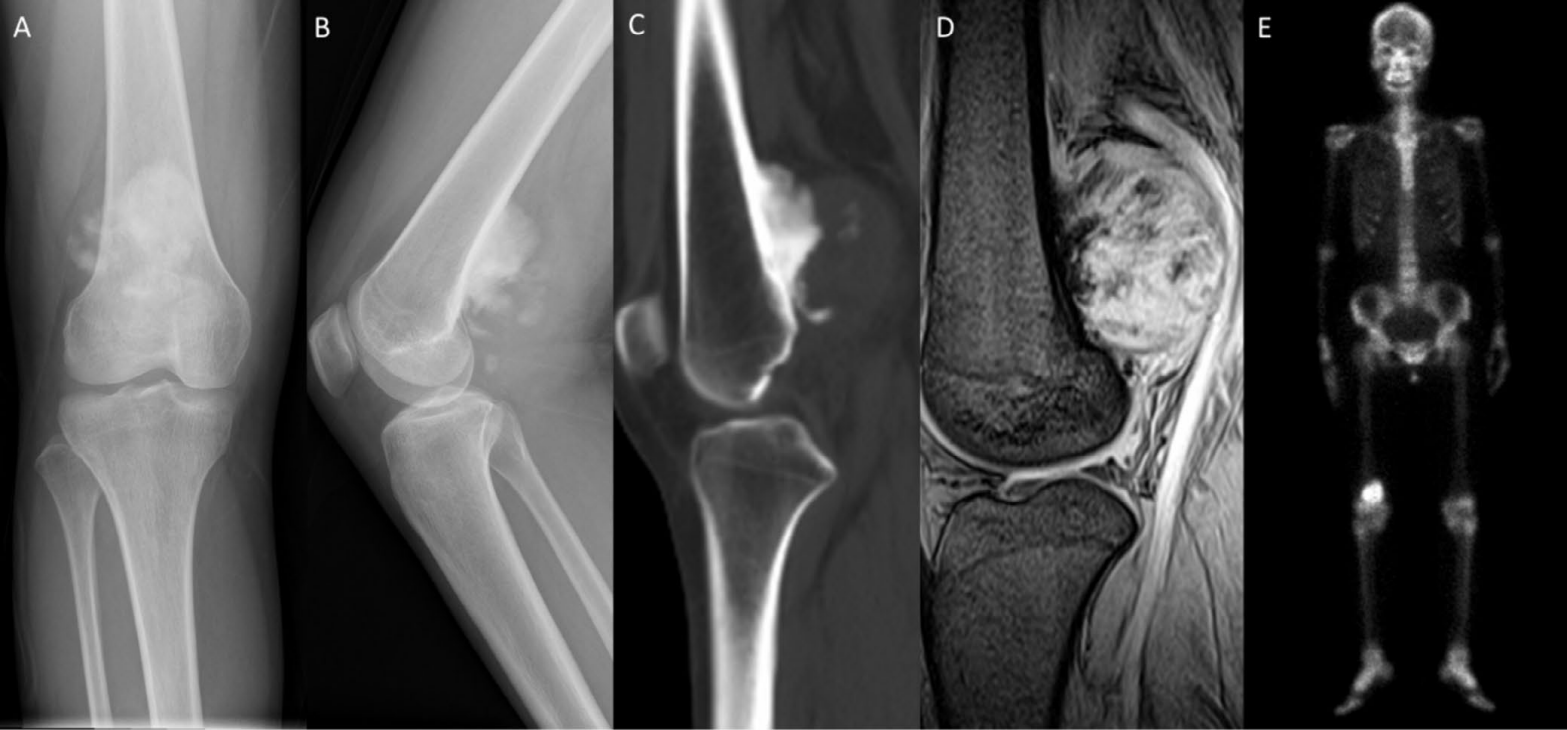
Preoperative anteroposterior (A) and lateral (B) X‐ray, CT (C), MRI (D), and SPECT scan (E) of a 30‐year‐old patient with highly differentiated and low‐grade parosteal osteosarcoma involving the right femur.

A comprehensive preoperative imaging assessment was performed for all patients, incorporating knee X‐rays, femoral 3D‐CT scans, knee MRI, and single‐photon emission computed tomography (SPECT) (Figure 1). Additionally, thin‐layer chest CT scans were conducted to detect potential metastatic spread to the lungs. Each patient underwent a biopsy before definitive surgery. This study was performed in compliance with the principles of the Declaration of Helsinki and was approved by the Ethics Committee (2025‐922).

### 2.2 Personalized Implant Design and Fabrication

Each prosthesis was tailored using an electron beam melting technique (ARCAM Q10plus) and fabricated by Chunli Co. Ltd., Beijing, China. Preoperative femoral CT data were utilized to create virtual 3D femur models in Mimics V20.0 software (Materialize Corp., Belgium). Tumor borders and osteotomy planes were determined via preoperative CT and MRI. The preliminary prosthesis shape was designed using Boolean operations on mirrored 3D models of the normal part and the post‐osteotomy anatomical model.

The customized 3DPSP was fabricated from medical‐grade Ti‐6Al‐4V ELI (ASTM F136) and consisted of solid structural plates combined with porous structural supports, featuring an average porosity of 75% and an optimized pore size ranging from 300 to 900 μm. Biomechanical characterization of this integrated porous architecture revealed a compressive strength of 85–110 MPa, which meets the mechanical demands of the distal femoral cortex. The 3D printing‐mediated porous architecture reduced the elastic modulus to 1–7 GPa, closely matching the native mechanical behavior of human bone and thereby facilitating physiological bone remodeling. The overall shape was elongated and trapezoidal, with two circular holes at the top and multiple regularly arranged holes in the main body. This allowed the prosthesis to be securely fixed to the femoral cortex with screws, ensuring stability during use. The final prosthesis data were imported into an electron beam melting system for precision manufacturing.

### 2.3 Surgical Techniques

**FIGURE 2:**
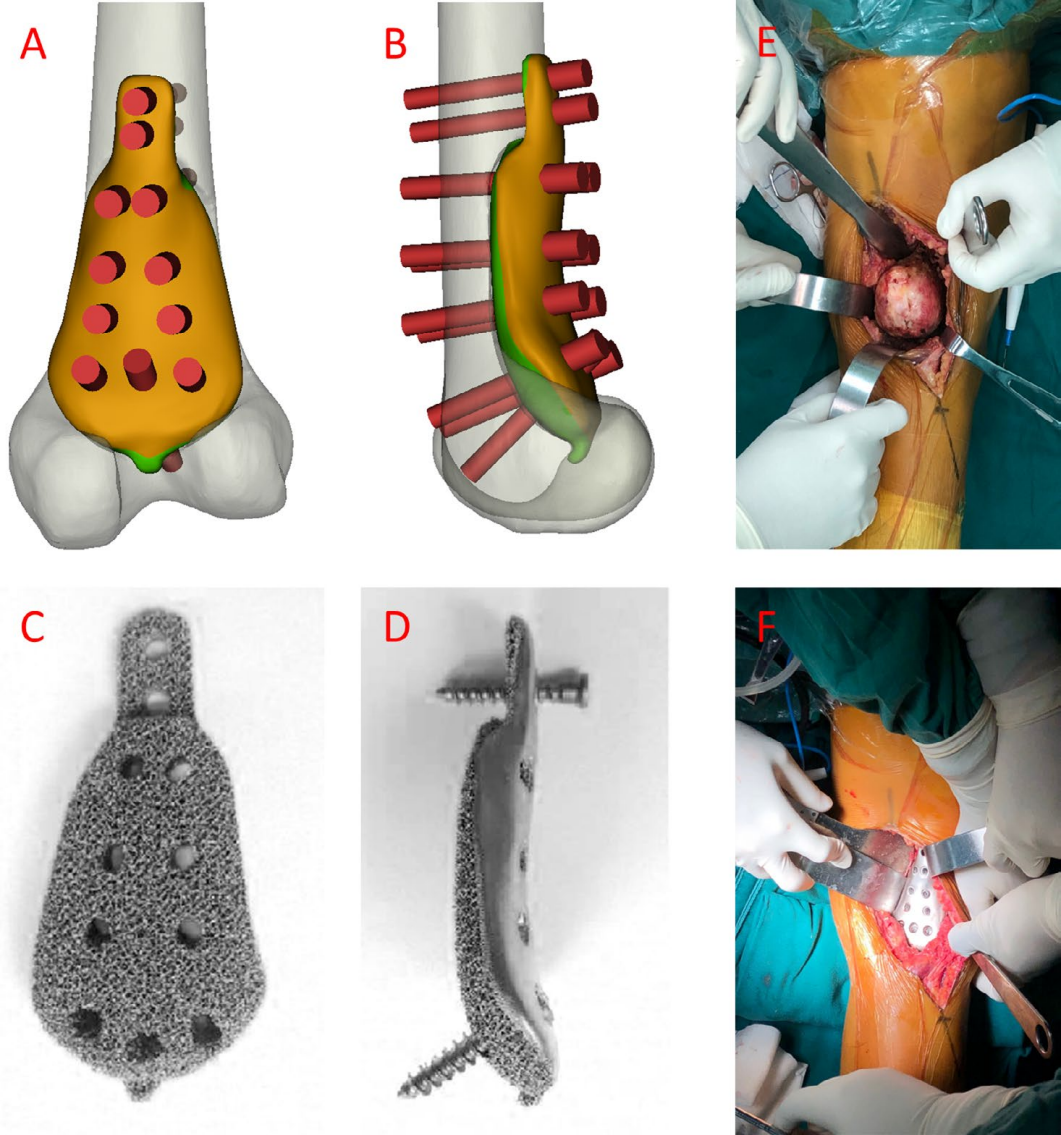
Photographs of the 3DPSP: (A, B) Pre‐operation simulation of tumor resection and prosthesis implant. (C, D) The 3D‐printed personalized implant with porous titanium surface. (E, F) Intraoperative pictures of prosthesis implantation.

**FIGURE 3:**
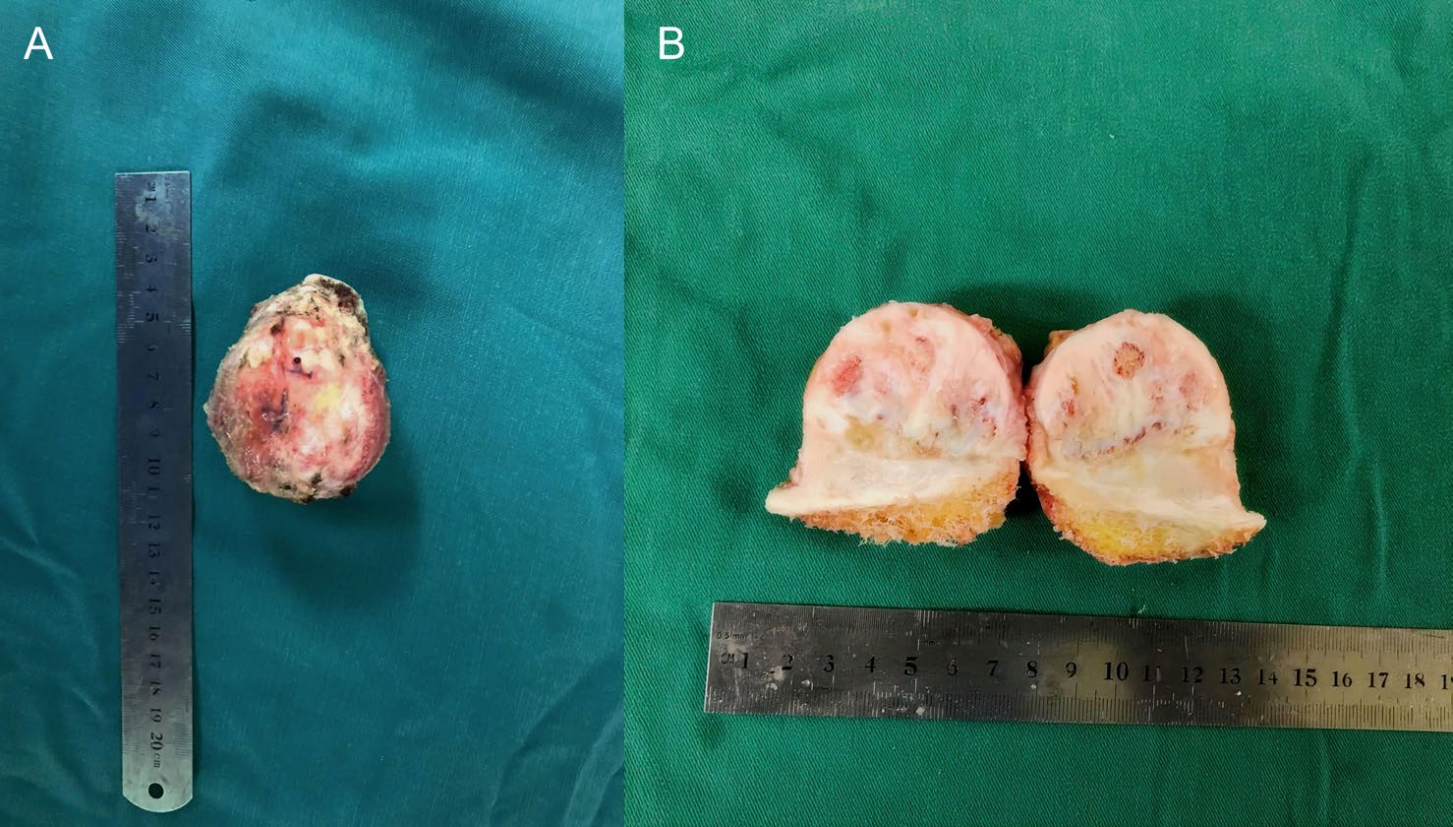
Macroscopic appearance of the resected tumor. (A) Gross specimen of the distal femur parosteal osteosarcoma; (B) Longitudinal section of the specimen.

All surgeries were performed by the senior surgeon Chongqi Tu. For tumors on the posterior femur, a single‐incision posterolateral approach was used for small tumors, while a double‐incision approach was used for larger ones. The semitendinosus, semimembranosus, and long head of the biceps femoris were carefully dissected to expose the femoral cortex. Adjacent tumor vessels were carefully separated and protected with retractors. A periosteal elevator was used to strip the surrounding soft tissue from the bone approximately 3–5 cm above and below the tumor site. Once the surgical site was clearly visible, a multi‐plane osteotomy was performed. Trial prostheses were used to ensure an optimal fit with the bone defects, which were thoroughly inspected and trimmed after grafting. After confirming the correct placement of the porous titanium prosthesis, screws were carefully inserted to secure the implant (Figure 2). Figure 3 shows the resected tumor specimens obtained during surgery. The 3DPSP was rigidly secured using 9–11 titanium screws measuring 5.0 mm in diameter and 40 mm in length. These screws were uniformly distributed and inserted perpendicularly to the prosthesis–bone interface to ensure optimal load sharing and bicortical engagement.

For patients undergoing DFR, an extended anteromedial approach was typically utilized. The tumor and involved soft tissues were carefully dissected en bloc. Major neurovascular structures, including the popliteal vessels and sciatic nerve branches, were systematically isolated and protected. Following the planned osteotomy, the affected distal femur was completely resected. The femoral and tibial medullary canals were sequentially reamed. Appropriate modular distal femoral tumor endoprostheses were then assembled and implanted. Cementless biological stems were primarily used for femoral fixation. After confirming satisfactory joint stability and range of motion, the extensor mechanism and surrounding muscles were meticulously reconstructed to ensure adequate soft‐tissue coverage. Meticulous hemostasis was achieved, and closed suction drains were placed prior to layered wound closure.

### 2.4 Postoperative Management and Follow‐Up

For patients in the 3DPSP group, rehabilitation was initiated with early ankle pumps and knee flexion‐extension exercises. Protected weight‐bearing was encouraged beginning 2 weeks postoperatively, with progression to full weight‐bearing, walking, and stair‐climbing as tolerated. In the DFR group, a more conservative rehabilitation protocol was implemented. The knee was immobilized in a brace initially, with weight‐bearing restrictions typically maintained for 6–8 weeks to protect the extensor mechanism reconstruction and soft tissue envelope; gait training with assistive devices was then gradually introduced.

For all patients, follow‐up visits were scheduled monthly for the first 3 months and quarterly thereafter. Clinical and imaging assessments were performed at each visit. Standard anteroposterior and lateral radiographs of the knee were obtained to evaluate implant alignment, position, and the presence of radiolucent lines. CT with metal artifact reduction technology (Shimadzu T‐SMART) was utilized to specifically assess osseointegration at the bone‐implant interface. Functional outcomes for both groups were evaluated using the Musculoskeletal Tumor Society (MSTS) 93 scoring system at designated intervals, with the primary endpoint defined as the functional outcome assessed by the MSTS score at the final follow‐up.

### 2.5 Statistical Analysis

All statistical analyses were performed using IBM SPSS Statistics software, version 26 (IBM SPSS, Armonk, NY, USA). Continuous data were presented as mean ± standard deviation (SD). Given the small sample size of the current study, which limits the reliability of the Shapiro–Wilk test for assessing normality, nonparametric methods were preferentially employed; thus, continuous variables were compared between groups using the Wilcoxon rank‐sum test (Mann–Whitney *U* test). Categorical variables were summarized as counts and percentages, with between‐group comparisons performed exclusively using Fisher's exact test to account for the small expected frequencies. All statistical tests were two‐sided, and a *p* < 0.05 was considered statistically significant.

## 3 Results

A total of 12 PAOS patients were enrolled in this study (Table 1). Baseline demographics demonstrated comparable mean ages between the 3DPSP and DFR cohorts (29.2 ± 4.3 vs. 29.8 ± 13.2 years), although gender distribution varied. Oncological characteristics were uniform, with all 12 patients achieving wide surgical resection margins. Perioperatively, the 3DPSP group demonstrated superior efficiency. Compared to the DFR group, the 3DPSP reconstruction required a shorter mean operation time (2.32 ± 0.38 vs. 3.05 ± 0.31 h) and resulted in substantially less intraoperative blood loss (175.0 ± 82.2 vs. 400.0 ± 141.4 mL), corresponding to mean resection lengths of 10.75 ± 1.80 cm and 14.60 ± 2.94 cm, respectively. The mean postoperative follow‐up was 46.3 ± 8.7 months for the 3DPSP group and 37.8 ± 13.8 months for the DFR group. The lower mean MSTS scores observed in the DFR group compared to the 3D‐printed group may be primarily attributed to the greater surgical trauma, more extensive bone loss, and the necessary sacrifice of crucial ligamentous structures associated with DFR (Tables 2 and 3).

**TABLE 1:** Demographics, clinical characteristics of patients.

Patients	Gender	Age (years)	Site	Diagnosis	Enneking stage	Follow‐up (months)	Margin
DFR	F	26	Femur	PAOS	IB	40	Wide
	F	24	Femur	PAOS	IB	32	Wide
	M	16	Femur	PAOS	IB	22	Wide
	M	25	Femur	PAOS	IB	57	Wide
	M	54	Femur	PAOS	IB	26	Wide
	M	34	Femur	PAOS	IB	50	Wide
3DPSP	F	31	Femur	PAOS	IB	39	Wide
	F	28	Femur	PAOS	IB	59	Wide
	F	30	Femur	PAOS	IB	52	Wide
	M	22	Femur	PAOS	IB	48	Wide
	F	35	Femur	PAOS	IB	45	Wide
	M	29	Femur	PAOS	IB	35	Wide

Abbreviations: 3DPSP: 3D‐printed strut‐type prosthesis; DFR: distal femoral replacement; F: female; M: male; PAOS: parosteal osteosarcoma.

**TABLE 2:** Clinical outcomes of patients undergoing hemicortical resection reconstructed with 3DPSP versus DFR.

Patients	Cortical circumference (%)	Resection length (cm)	Operation time (h)	ROM	Blood loss (mL)	MSTS score	Complications	Oncological status
DFR	50	15.6	3	0–100	600	29	Aseptic loosening	Local recurrence
	55	12.4	2.5	0–105	300	21	NA	NED
	45	10.5	3.2	0–120	200	30	NA	NED
	45	15.2	3	0–115	400	27	NA	NED
	40	19.1	3.4	0–110	500	23	NA	NED
	40	14.8	3.2	0–105	400	22	NA	NED
3DPSP	30	9.9	2	0–130	150	30	NA	NED
	50	11.9	3	0–110	300	26	NA	NED
	25	8.9	2.2	0–125	100	30	NA	NED
	40	10.4	2.5	0–130	250	29	NA	NED
	30	9.6	2	0–125	100	28	NA	NED
	35	13.8	2.2	0–120	150	27	NA	NED

Abbreviations: 3DPSP: 3D‐printed strut‐type prosthesis; DFR: distal femoral replacement; MSTS: musculoskeletal tumor society; NA: none; NED: no evidence of diseases; ROM: range of motion.

**TABLE 3:** Comparison of baseline characteristics and clinical outcomes between the 3DPSP and DFR groups.

Characteristic	Overall *N* = 12	3DPSP *N* = 6	DFR *N* = 6	*p*‐Value	Statistic
Gender				0.567	*χ* ^2^ = 0.333
F	6 (50%)	4 (67%)	2 (33%)		
M	6 (50%)	2 (33%)	4 (67%)		
Age (years)	29.500 ± 9.327	29.167 ± 4.262	29.833 ± 13.152	0.589	*W* = 22
Site					
Femur	12 (100%)	6 (100%)	6 (100%)		
Diagnosis					
PAOS	12 (100%)	6 (100%)	6 (100%)		
Enneking stage					
IB	12 (100%)	6 (100%)	6 (100%)		
Follow‐up (months)	42.083 ± 11.836	46.333 ± 8.710	37.833 ± 13.747	0.310	*W* = 25
Margin					
Wide	12 (100%)	6 (100%)	6 (100%)		
Cortical circumference (%)	40.417 ± 9.160	35.000 ± 8.944	45.833 ± 5.845	0.052	*W* = 5.5
Resection length (cm)	12.675 ± 3.075	10.750 ± 1.801	14.600 ± 2.943	0.015	*W* = 3
Operation time (h)	2.683 ± 0.506	2.317 ± 0.382	3.050 ± 0.308	0.015	*W* = 2.5
Blood loss (mL)	287.500 ± 161.139	175.000 ± 82.158	400.000 ± 141.421	0.016	*W* = 2.5
MSTS score	26.833 ± 3.215	28.333 ± 1.633	25.333 ± 3.830	0.225	*W* = 26

Abbreviations: 3DPSP: 3D‐printed strut‐type prosthesis; DFR: distal femoral replacement; F: female; M: male; MSTS: musculoskeletal tumor society; PAOS: parosteal osteosarcoma.

**FIGURE 4:**
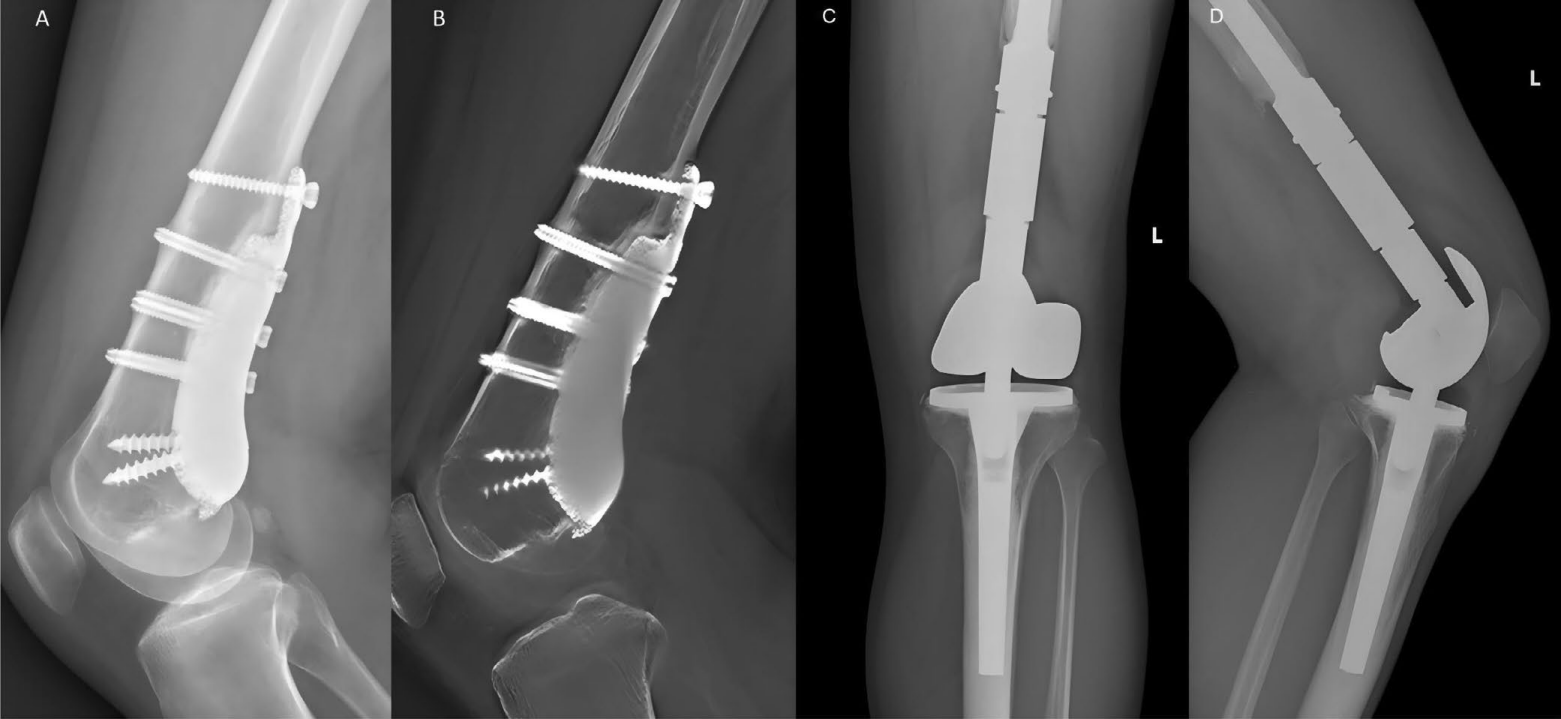
Postoperative radiographs. (A) The X‐rays demonstrate the proper position of the 3DPSP; (B) T‐SMART showed no significant loosening at the bone‐prosthesis interface. (C) Anteroposterior and (D) lateral radiographs of a representative patient from the DFR control group at postoperative follow‐up, showing the implanted distal femoral endoprosthesis.

**FIGURE 5:**
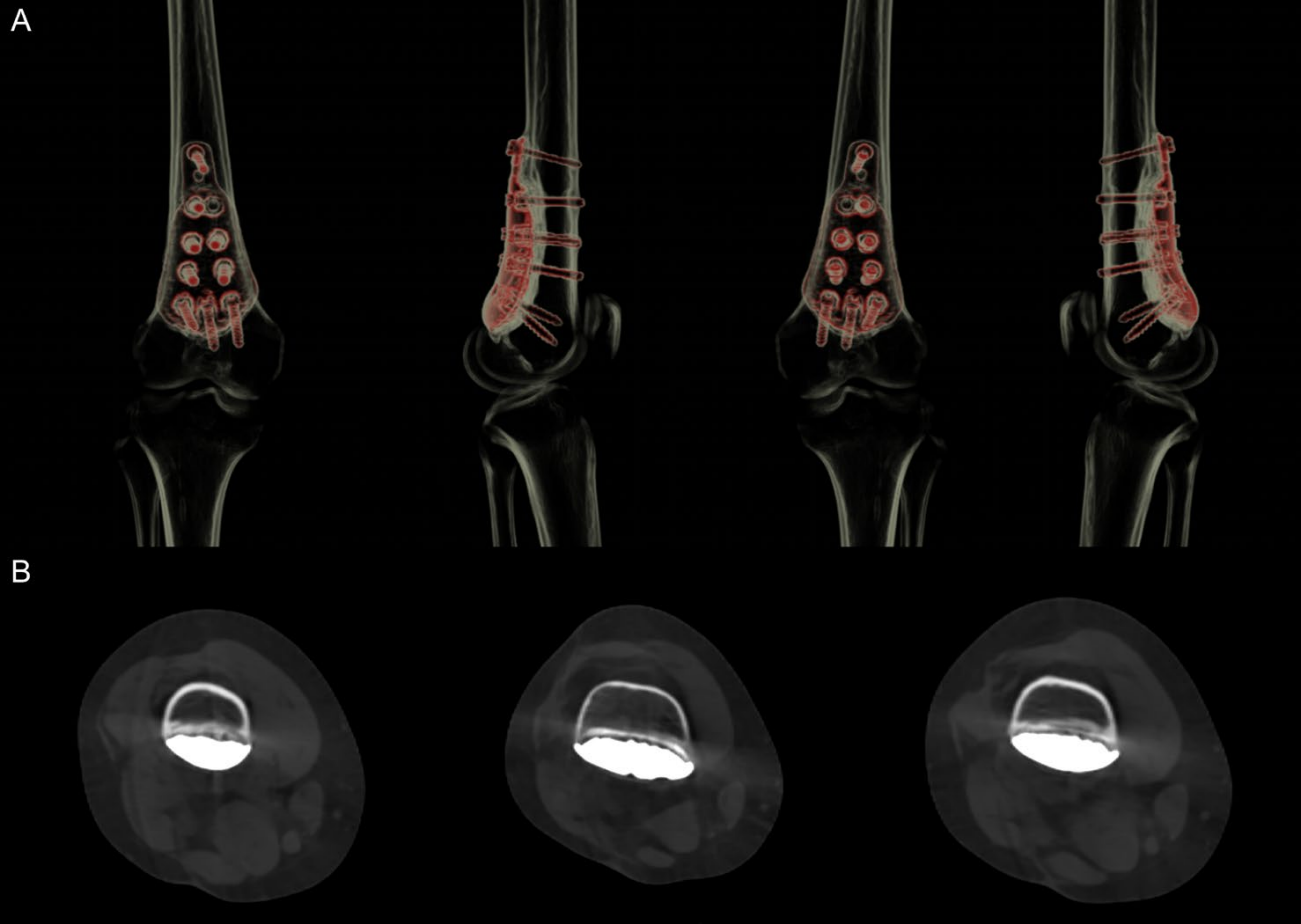
Postoperative radiographic and 3D CT evaluation of a customized 3DPSP. (A) 3D volume‐rendered CT reconstruction images of the distal femur demonstrating four different anatomical perspectives: posterior, lateral, anterior, and medial views (from left to right). The prosthesis and bicortical fixation screws are highlighted in red to facilitate visualization of their precise anatomical fit and screw positioning relative to the host bone. (B) Representative axial CT images across three different sequential levels (from left to right): proximal, midpoint, and distal. These cross‐sectional views show the interaction between the porous implant and the femoral cortex at different points along the femoral shaft.

Postoperative X‐rays and CT scan confirmed the precise alignment of the 3DPSP with the hemicortical bone defects. T‐SMART imaging showed good bone‐to‐implant contact and uniform bone density around the implants (Figures 4 and 5).

**FIGURE 6:**
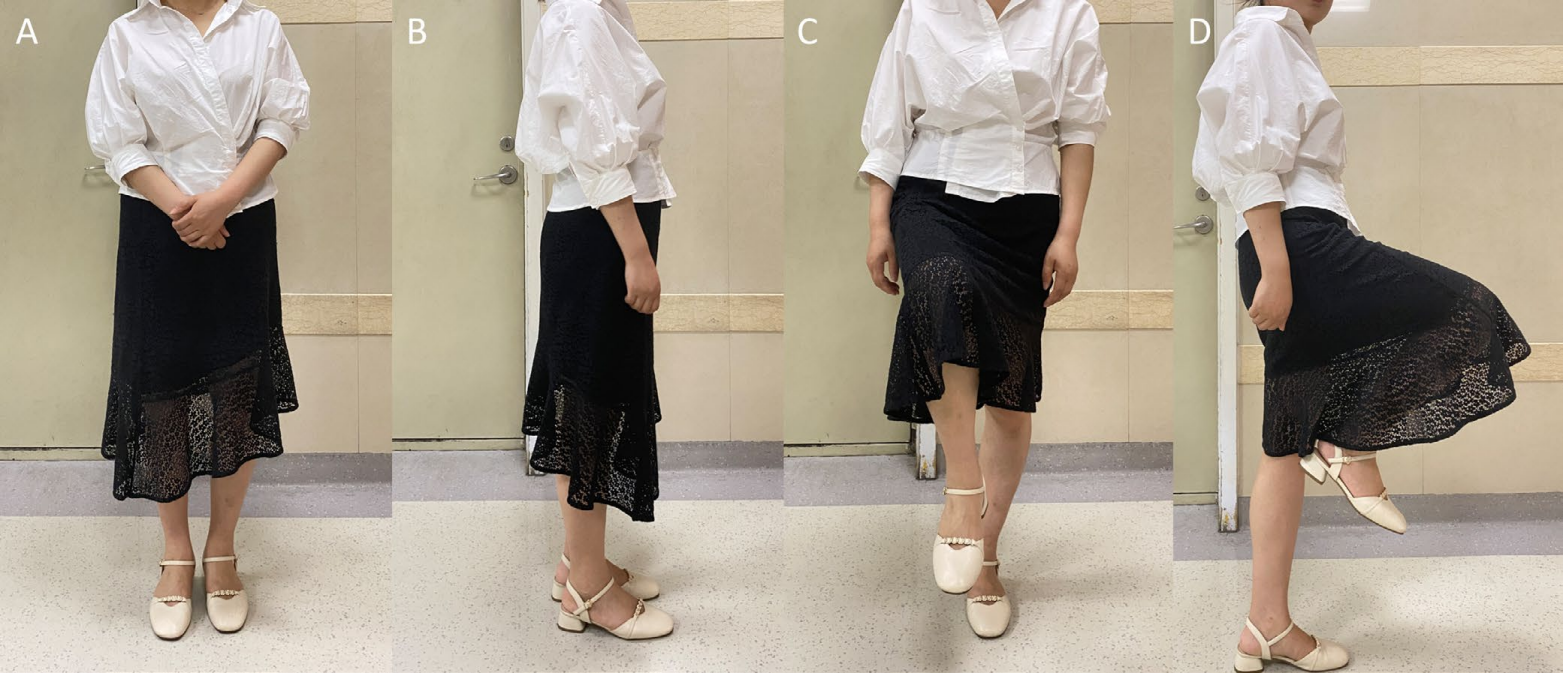
Four years post‐operation, the patient's knee function was favorable. (A) Standing position, anterior view; (B) standing position, lateral view; (C) the patient could stand on the affected limb without pain; (D) knee flexion was normal.

In the 3DPSP group, the average MSTS score was 28.3 ± 1.6 (range, 26–30), and the average range of motion of the knee was 0°–123° degrees at the final follow‐up. No patients required walking aids (Figure 6). During the follow‐up, there was no instance of infection, implant fractures, periprosthetic fracture, nerve palsy, or vascular incidents (Table 2). In contrast, one patient in the DFR group experienced local tumor recurrence and femoral component loosening at 16 months postoperatively.

## 4 Discussion

This study demonstrates that a novel 3DPSP provides a reliable hemicortical reconstruction option for patients with PAOS of the distal femur. At a mean mid‐term follow‐up of 46.3 months, the 3DPSP cohort exhibited satisfactory oncological control with no local recurrences, excellent functional recovery (mean MSTS score 28.3, knee flexion 0°–123°), and favorable prosthesis–bone osseointegration without aseptic loosening or implant‐related fractures. When compared with a concurrent DFR group, the 3DPSP reconstruction was associated with significantly shorter operative time, less intraoperative blood loss, and superior MSTS scores, highlighting the clinical benefits of this joint‐preserving strategy.

### 4.1 Clinical Outcomes and Comparison With Distal Femoral Replacement

We observed promising results with a mean follow‐up period of 46.3 ± 8.7 months (range: 35–59 months), demonstrating satisfactory limb function, high prosthesis stability, and a low complication rate. Specifically, the mean MSTS score was 28.3 ± 1.6, and the knee flexion range was 0°–123°. These outcomes are comparable to satisfactory functional and oncological results reported in other studies [22, 23, 24, 25]. Specifically, these studies involved hemicortical resection for distal femur PAOS. Liu et al. observed positive outcomes and low complication rates in 13 PAOS patients. These patients underwent wide resection and reconstruction using pasteurized autografts and internal fixation. They reported a mean MSTS score of 88.6% (range: 80%–100%) [26]. Traditional biological reconstructions often require prolonged immobilization to facilitate bone union. Unfortunately, this delays functional recovery. For instance, Liu et al. [26] and Chen et al. [11] reported that patients were permitted partial weight‐bearing only 3 months postoperatively. Full weight‐bearing was delayed until there was evidence of bone union. Similarly, Agarwal et al. [27] utilized a long‐leg cast for immobilization for up to 6 weeks. In contrast, our 3DPSP provides immediate mechanical stability through its titanium strut. This allowed patients to begin weight‐bearing exercises as tolerated just 2 weeks after surgery. The 3DPSP avoids joint stiffness and muscle atrophy caused by prolonged immobilization. Furthermore, it significantly shortens the time required for patients to return to normal daily activities.

Prior research has shown that fractures, infections, and incomplete resections are the main causes of complications after hemicortical resection. Notably, fractures are among the most common causes of these complications, with an incidence ranging from 10% to 18% [28]. This high fracture rate is typically attributed to the insufficient mechanical strength or delayed union often seen with biological grafts. In our study, the operation duration was 2.3 ± 0.4 h with an average volume of blood loss of 175 ± 82.2 mL. Beyond providing superior mechanical stability, the 3DPSP strategy significantly reduced surgical trauma. In our control cohort, the DFR group exhibited outcomes consistent with prior literature regarding DFR, characterized by longer operation times (3.05 ± 0.31 h), increased blood loss (400.0 ± 141.4 mL), and lower functional scores (MSTS 25.3 ± 3.8), primarily due to the extensive osteotomy and extensive soft tissue dissection required for massive endoprostheses. When compared with the study by Zhang et al. using pasteurized autografts, our method maintained comparable resection lengths (10.8 cm vs. 9.5 cm) and operation times (2.3 h vs. 2.5 h), yet demonstrated a marked reduction in blood loss compared to their reported average of 1000 ± 350 mL [29]. This improvement is likely attributable to the personalized nature of the 3DPSP; unlike traditional grafts that necessitate tedious intraoperative manual shaping and repeated fitting, the customized prosthesis simplifies the surgical workflow, thereby significantly minimizing unnecessary invasive maneuvers and soft tissue stripping. No fractures, infections, or recurrences were observed at the final follow‐up. The absence of fractures might be attributed to several factors. Firstly, the cortical bone resection did not exceed 50% of the circumference. Secondly, the personalized implants perfectly matched the bone defects, conforming to anatomical characteristics and facilitating mechanical conduction. Crucially, while the DFR group necessitated the complete sacrifice of the knee joint and its stabilizing ligaments, the 3DPSP group preserved the majority of the host bone and joint integrity, resulting in a significantly higher MSTS score (28.3 ± 1.6 vs. 25.3 ± 3.8). Compared to traditional endoprosthetic DFR, the smaller volume of the implants and better soft tissue coverage contributed to a reduced risk of infection. In conclusion, 3DPSP reconstruction is an innovative approach that highly preserves joint function, promotes osseointegration, and reduces postoperative complications and recurrence rates.

### 4.2 Osseointegration and Mechanical Stability

No aseptic loosening of the prosthesis occurred in this study, and all radiographic images demonstrated evidence of bone proliferation at the bone‐prosthesis interface, indicating good osseointegration. These favorable outcomes are likely attributable to the material characteristics of the implant. The 3DPSP, characterized by its specific pore size and porosity on the porous titanium surface, demonstrates superior biocompatibility. Solid titanium has an elastic modulus significantly higher than human bone tissue. Adding a porous structure significantly reduces the stiffness of the material, making it almost an order of magnitude lower and thereby promoting bone conduction and integration. Torres‐Sanchez et al. found that a porous scaffold with a 300–900 μm pore size and 70% porosity can effectively mimic trabecular bone [30]. In this research, we designed and manufactured 3DPSP based on these parameters. Previously, autografts or allografts were employed for hemicortical defect reconstruction, a method that requires considerable effort and time from surgeons to precisely shape the graft to fit perfectly with the bone defect area [31]. This manual approach often resulted in unsatisfactory accuracy of the grafts. Additionally, patients risked graft resorption and nonunion [32]. The personalized 3D‐printed implants in this study offer a precise fit for hemicortical defects and reduce the need for intraoperative adjustments compared to traditional bone grafting. The application of the 3DPSP effectively mitigates these risks through precise anatomical matching and optimized material properties.

### 4.3 Broader Applicability of 3D‐Printed Strut Prostheses

Beyond the femur, 3D‐printed supportive prostheses show great potential in reconstructing other challenging anatomical sites. In the lower extremity, the tibia endures massive axial loads [33]. Custom 3D‐printed implants can precisely match tibial bone defects [34]. This anatomical match may provide immediate mechanical stability and allow for early weight‐bearing. For the upper limb, the primary biomechanical requirement is complex rotational mobility rather than pure load‐bearing [35, 36]. 3D‐printed prostheses can accurately reconstruct joint congruity and specific soft‐tissue attachment sites. This precise reconstruction effectively preserves essential rotational functions of the arm. Furthermore, pelvic tumor resections create extreme reconstructive challenges due to highly irregular bone geometry and complex force transmission [37]. Patient‐specific 3D‐printed pelvic prostheses can perfectly conform to the residual bone contour. This custom fit successfully restores the pelvic ring integrity. It also ensures proper load transfer from the spine to the lower extremities [38].

### 4.4 Strengths and Limitations

This study introduces a novel 3D‐printed strut‐type prosthesis for hemicortical reconstruction of the distal femur. The implant promotes osseointegration and provides immediate mechanical stability that permits early weight‐bearing. At a mean follow‐up of 46 months, the 3DPSP cohort achieved excellent functional recovery (mean MSTS score 28.3, knee flexion 0°–123°) with no local recurrence, implant fracture, or aseptic loosening. Compared with distal femoral replacement, the procedure was associated with shorter operative time, significantly less intraoperative blood loss, and accelerated postoperative rehabilitation owing to early mobilization.

However, the procedure remains technically demanding. Surgical exposure is particularly challenging for posterior distal femoral lesions because of the close relationship between the tumor, popliteal vessels, and surrounding soft tissues. Accurate multi‐plane osteotomy, preservation of cortical continuity, meticulous soft tissue handling, and stable screw fixation perpendicular to the prosthesis–bone interface are essential for achieving immediate mechanical stability and preventing postoperative micromotion. In our experience, careful patient selection, strict control of cortical resection extent, and accurate prosthesis positioning are key factors contributing to favorable oncological safety, stable fixation, and satisfactory functional recovery.

This study has several limitations. First, the retrospective design and small sample size from a single institution inevitably limit the statistical power and generalizability of the findings. Second, selection bias may exist because the indication for 3DPSP reconstruction was restricted to localized lesions without articular involvement, whereas patients undergoing DFR generally presented with more extensive tumor burden. Third, the follow‐up duration was relatively limited and insufficient to fully evaluate long‐term prosthesis survival, oncological safety, and late complications such as aseptic loosening or stress shielding. Finally, although favorable early osseointegration and mechanical stability were observed, comprehensive biomechanical validation and larger multicenter prospective studies are still required to further confirm the long‐term reliability and broader applicability of this novel prosthetic strategy.

## 5 Conclusion

In this study, we designed a novel 3DPSP to reconstruct the hemicortical defect after distal femur PAOS resection. The mid‐term follow‐up result demonstrated that this prosthesis could be an effective reconstruction option for distal femur PAOS patients because of the restored limb function and favorable bone‐prosthesis integration. Nevertheless, given the small sample size and limited application in the current study, further long‐term observation of this novel prosthesis is warranted.

## Author Contributions

**Yitian Wang:** investigation, conceptualization, visualization, project administration, formal analysis, writing – review and editing. **Jianhua Mu:** conceptualization, investigation, funding acquisition, formal analysis, supervision, data curation, resources, writing – review and editing. **Han Liu:** investigation, conceptualization, formal analysis, data curation, software, methodology, validation, writing – review and editing. **Minxun Lu:** investigation, validation. **Zhuangzhuang Li:** investigation, funding acquisition. **Fan Tang:** funding acquisition, visualization. **Yi Luo:** funding acquisition, formal analysis. **Xuanhong He:** investigation, funding acquisition, visualization, validation, project administration. **Chongqi Tu:** writing – review and editing, funding acquisition, investigation, validation, project administration, formal analysis, software. **Li Min:** investigation, funding acquisition, writing – review and editing, project administration, formal analysis. **Yong Zhou:** conceptualization, validation.

## Funding

This work was supported by National Natural Science Foundation of China (82302690).

## Ethics Statement

This study was performed in accordance with the Declaration of Helsinki as revised in 2008 and was approved by the Ethics Committee of West China Hospital. The patients signed the informed consent form before surgery.

## Consent

Informed consent was obtained from all participants included in the study.

## Conflicts of Interest

The authors declare no conflicts of interest.

## Supporting information


**Video S1:** Gait.

**Video S2:** Knee range of motion.

**Video S3:** Squat (lateral view).

**Video S4:** Squat anterior view.

**Table S1:** os70360‐sup‐0001_Table_S1.xlsx.

## Data Availability

The data that support the findings of this study are available on request from the corresponding author. The data are not publicly available due to privacy or ethical restrictions.
